# Frailty Intervention Trial (FIT)

**DOI:** 10.1186/1471-2318-8-27

**Published:** 2008-10-13

**Authors:** Nicola Fairhall, Christina Aggar, Susan E Kurrle, Catherine Sherrington, Stephen Lord, Keri Lockwood, Noeline Monaghan, Ian D Cameron

**Affiliations:** 1 The George Institute for International Health, The University of Sydney, Sydney, Australia; 2 Faculty of Nursing and Midwifery, The University of Sydney, Sydney, Australia; 3 Rehabilitation and Aged Care Services, Hornsby Ku-ring-gai Hospital, Sydney, Australia; 4 Prince of Wales Medical Research Institute, University of New South Wales, Sydney, Australia; 5 Rehabilitation Studies Unit, Faculty of Medicine, The University of Sydney, Sydney Australia

## Abstract

**Background:**

Frailty is a term commonly used to describe the condition of an older person who has chronic health problems, has lost functional abilities and is likely to deteriorate further. However, despite its common use, only a small number of studies have attempted to define the syndrome of frailty and measure its prevalence. The criteria Fried and colleagues used to define the frailty syndrome will be used in this study (i.e. weight loss, fatigue, decreased grip strength, slow gait speed, and low physical activity). Previous studies have shown that clinical outcomes for frail older people can be improved using multi-factorial interventions such as comprehensive geriatric assessment, and single interventions such as exercise programs or nutritional supplementation, but no interventions have been developed to specifically reverse the syndrome of frailty.

We have developed a multidisciplinary intervention that specifically targets frailty as defined by Fried et al. We aim to establish the effects of this intervention on frailty, mobility, hospitalisation and institutionalisation in frail older people.

**Methods and Design:**

A single centre randomised controlled trial comparing a multidisciplinary intervention with usual care. The intervention will target identified characteristics of frailty, functional limitations, nutritional status, falls risk, psychological issues and management of chronic health conditions. Two hundred and thirty people aged 70 and over who meet the Fried definition of frailty will be recruited from clients of the aged care service of a metropolitan hospital. Participants will be followed for a 12-month period.

**Discussion:**

This research is an important step in the examination of specifically targeted frailty interventions. This project will assess whether an intervention specifically targeting frailty can be implemented, and whether it is effective when compared to usual care. If successful, the study will establish a new approach to the treatment of older people at risk of further functional decline and institutionalisation. The strategies to be examined are readily transferable to routine clinical practice and are applicable broadly in the setting of aged care health services.

**Trial Registration:**

Australian New Zealand Clinical Trails Registry: ACTRN12608000250336.

## Background

Frailty is a term in common use among health care professionals. It is a term often used to label the condition of an older person who has health problems, has lost functional abilities and is likely to deteriorate further. It is thus a syndrome, describing a health state that could occur as the result of a number of underlying health conditions. As a summary term it has some utility, but little practical clinical or scientific value unless it is adequately defined.

Rockwood and colleagues [1] describe the concept of frailty as a 'multidimensional syndrome of loss of reserves (energy, physical ability, cognition, health) that gives rise to vulnerability'. The clinical conceptualisation of frailty has been described as an observable physical and functional decline in the body associated with physiological changes during later life [2], which is accompanied by social and emotional experiences [3]. Clinical research on frailty has enabled the provision of care to the older population to target those most at risk [4] and in need [5], and to allocate appropriate services and resources [6].

However, frailty does not have a precise definition [7] and is not a specific diagnosis [8]. It is seen as an increasingly common condition in older people but is not an inevitable part of ageing. Many older people are not frail and never become frail, and frailty is also observed in younger people. It is not synonymous with disability as many disabled people are not frail. However, frailty results in disability, and it usually indicates a person at increased risk for morbidity and mortality [9,10].

There have been a number of attempts to define frailty. It has been described in terms of a syndrome, characterised by decreased reserve and resistance to stressors, resulting from cumulative declines across multiple physiological systems that are close to or past symptomatic clinical failure [11]. Rockwood [12] proposed that the essential feature of frailty is the notion of risk due to instability. Consequently, the frail person is at increased risk of disability and death. Frailty has been described as a "pre-death" syndrome [13], and it has been shown to be the commonest trajectory at the end of life, and to be associated with stroke, Alzheimer's Disease and other dementias, delirium, Parkinson's Disease, hip fracture, incontinence, pneumonia, dehydration, syncope, lower limb cellulitis [14]. Bortz [8] postulated that frailty is the result of early disease in multiple systems resulting in impaired muscle strength, mobility, balance and endurance. He considered that active intervention should be able to reverse much of the state of frailty. The definition of frailty used in the FICSIT (Frailty and Injuries: Cooperative Studies of Intervention Techniques) trials is also focussed on strength, mobility, balance and endurance [15].

Following the Cardiovascular Health Study, Fried et al [16] formulated specific criteria that defined the frailty syndrome. These criteria offer an empirically derived and validated definition for frailty based on the presence of at least three or more defined characteristics. The presence of frailty, as defined by Fried et al, was shown to be independently predictive over a three year period of incident falls, worsening mobility, deteriorating function in activities of daily living, hospitalisation, and death. The Fried frailty characteristics are unexplained weight loss, muscle weakness, self-reported exhaustion, poor endurance (as demonstrated by slow walking speed) and low activity level.

In a United States community dwelling population, aged over 65 years, Fried et al [16] found that 7% of the population met the criteria for frailty. Prevalence of frailty increased with each five-year cohort, and was double in women compared to men. After analysis of data from 2762 older people, Fried et al [16] described three separate aspects of functioning: frailty, disability and co-morbidity. Of people who were frail, 73% concurrently experienced one or both of the other conditions.

Attempts have been made to improve clinical outcomes for frail older people, however no interventions have been developed to specifically reverse the syndrome of frailty. Previous intervention studies targeting frail older people have focussed on using general interventions such as comprehensive geriatric assessment [17-19] and rehabilitation models [20], with inconclusive effects on functional ability and well-being. A recent systematic review found community-based, multifactorial interventions reduce hospital admission in the frail population, but do not significantly benefit physical function in this group [21]. Specific interventions targeting physical activity have been shown to improve physical function [22,23], and intervention with nutritional supplements plus exercise has resulted in increased energy intake [23]. Pharmacological interventions [24-26] produced inconsistent results.

Major limitations of the intervention studies targeting frail older people include the absence of an agreed definition for the term 'frailty'which would allow the precise identification of appropriate patients [2], and the lack of combined intervention studies that evaluate individual needs and customise care to promote the development of function and quality of life in frail older people [21].

Many clients of aged care health services are frail. This study seeks to establish whether a multifactorial, multidisciplinary intervention specifically targeting frailty can be implemented in a general metropolitan hospital aged care service setting, and whether it is effective when compared with usual care.

We are seeking to establish the effect of the intervention on both frailty and mobility. Specifically the hypotheses are that the multifactorial, multidisciplinary intervention will improve performance on a frailty index score and on a mobility index score calculated from the Short Physical Performance Battery score. The secondary outcome measures include days of hospitalisation and residence in nursing care facilities, risk of falling, additional measures of mobility, depression and quality of life. In addition, the study will test whether the Fried definition of frailty can be applied readily in a clinical setting and the ability of an aged care health service to effectively provide the intervention.

If this frailty intervention is shown to be effective, there are major potential benefits to the frail older population generally in terms of decreased disability. Significant cost savings could flow to government and broader society if hospitalisation or institutionalisation can be avoided or postponed. The interventions being examined are readily transferable to routine clinical practice and can potentially be applied routinely in aged care services.

## Methods and Design

### Design Outline

**Figure 1:**
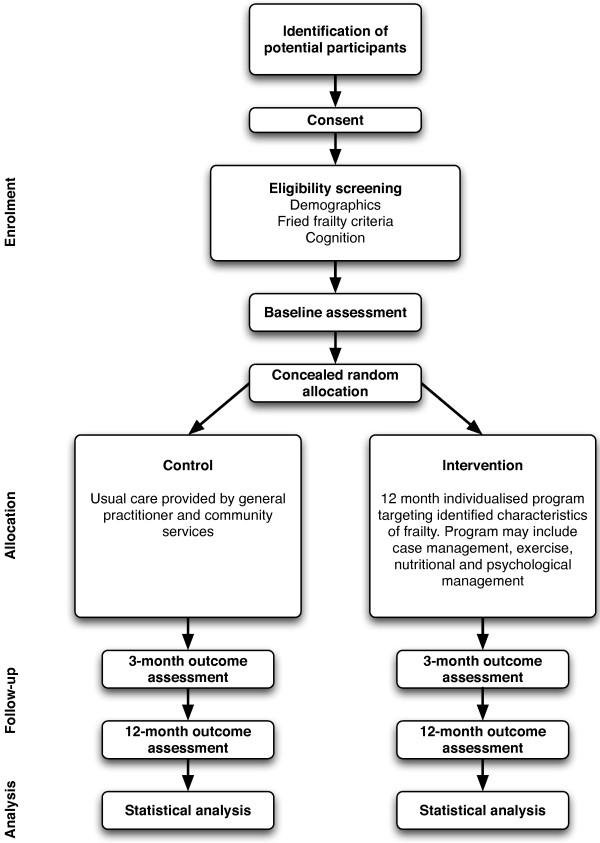
**Overview of the flow of participants through the Frailty Intervention Trial**. Adapted from the CONSORT diagram [27,28].

A single centre randomised controlled trial with a 12-month follow-up period will be conducted among 230 frail older persons (see Figure 1). A multidisciplinary intervention that targets identified characteristics of frailty will be compared with usual care. At the study site, usual care for non-institutionalised frail older people involves the General Practitioner (GP), community services and some home visiting programs to provide therapy.

### Recruitment and Eligibility

Eligible participants will be identified from older people (men and women aged 70 years or more) seen by clinicians working with the Division of Rehabilitation and Aged Care Services (DRACS) at Hornsby Ku-ring-gai Health Service (in urban northern Sydney, Australia). DRACS is a large clinical service that has programs operating in the community and in the hospital setting, and incorporates an Aged Care Assessment Team. Eligible participants will have completed their treatment program before being approached to enter the study. Once a person agrees to participate in the study informed consent will be sought, and if granted, the study nurse will screen for inclusion criteria.

The inclusion criteria are: adults aged 70 years or older with 3 or more of the Fried Frailty Criteria [16]; not usually living in a residential aged care facility; residing in the Hornsby or Ku-ring-gai local government areas (in Sydney, Australia); without severe cognitive impairment (defined as a Mini Mental State Examination (MMSE) score of 18 or less); not an ongoing client of DRACS and without an illness likely to be associated with a life expectancy of less than 12 months. People who do not meet all of the inclusion criteria or who do not consent will not be included. Residents of nursing care facilities are excluded from the study because one of the outcomes of interest is residence in a nursing care facility.

Participants satisfying the inclusion criteria will be individually randomised to either usual care from their general practitioner and community services, or to an individualised intervention designed to treat the identified components of frailty.

The Northern Sydney & Central Coast Health Human Research Ethics Committee has approved the study protocol – Research Protocol Number 0709-191M.

### Measurements and Procedures

Baseline measures will be assessed prior to randomisation. Outcomes will be assessed at three and twelve months by a researcher who has been blinded to group allocation. Participants will receive monthly calendars at the baseline assessment, with instructions to record the following events: falls; home visits by community services, nursing and allied health personnel; general practitioner appointments; use of hospital transport; receipt of delivered meals; hospitalisations and admissions to nursing care facilities. Participants will be asked to post the completed calendar to the blinded research personnel monthly. Telephone contact will be made with the participant if the calendar is not received, is completed incorrectly, or a fall, hospitalisation or admission to a nursing care facility, has occurred.

#### Baseline measures

Demographic detail and health information will be collected. Cognitive function will be assessed with the Mini Mental State Examination [29]. The number of previous falls and fractures will be recorded.

#### Primary outcomes

1. Frailty will be measured using the definition of the frailty syndrome by Fried and colleagues [16]. The tool measures five components of the frailty syndrome. One point is scored for each criterion met to specification, with participants meeting 3, 4 or 5 criteria defined as frail. To assess the effects of the intervention program, we will calculate an index score based on the five components. The components are:

a) Unintentional weight loss. At baseline the participant will be asked whether they have lost more than 4.5 kg unintentionally in the past year and their weight will be measured with scales. This criterion is positive if there is unintentional weight loss of more than 4.5 kg, or greater than 5% of body weight in the previous year.

b) Self-reported exhaustion. The participant will be read two statements from the Center for Epidemiologic Studies-Depression Scale [30]:

• "I felt that everything I did was an effort"

• "I could not get going"

The participant will then be asked how often in the last week he/she felt this way. 0 = rarely or none of the time, 1 = some or a little of the time (1–2 days), 2 = a moderate amount of the time (3–4 days), 3 = most of the time. A score of 2 or 3 is a positive response.

c) Weakness. Grip strength will be measured using a dynamometer (Saehan Dynamometer, model SH5001). The best of three attempts will be used. Using a simplification of the cut-off value for grip strength used by Fried et al [16], male participants who score 30 kg or less will be classified as having weak grip strength. Female participants with a score of 18 kg or less will be classified as having weak grip strength.

d) Slow walking speed. The time to walk four metres will be measured, with or without a walking aid. Those participants with a walking time of six seconds or more will be classified as having slow walking speed. The values used by Fried et al [16] have been modified slightly for ease of assessment. Cesari and colleagues [31] determined that older persons with a usual walking speed of one metre per second or less is indicative of poor health outcomes.

e) Low physical activity level. Participants will meet the criterion for physical inactivity if, in the past three months, they did not perform weight-bearing physical activity, spent more than four hours per day sitting, and went for a short walk once per month or less. This is a modification of the definition used by Cesari and colleagues [32].

2. Mobility will be assessed using the lower extremity continuous summary performance score [26] with data collected according to the methods described in the Short Physical Performance Battery [33]. The ability to stand (for 10 sec) with the feet together in the side-by-side, semi-tandem, and tandem positions, time to walk 4 metres, and time to rise from a chair and return to the seated position 5 times is examined. Performance for each component will be recoded as a score between 0 and 1 and summed using the methodology developed by Onder et al [26].

#### Secondary outcomes

1. Hospitalisations and admissions to nursing care facilities will be reported on the monthly calendars then confirmed from general practitioner or hospital records, or the appropriate facility.

2. Activities of daily living status will be measured by the Barthel Index [34], using the 100 point version.

3. The EQ-5D (EuroQol) will measure health related quality of life and provide utility weights to enable evaluation of cost-effectiveness [35]. The EQ-5D contains a visual analogue scale (0 to 100, representing dead to excellent health state) and five items: mobility; self-care; usual activities; pain/discomfort; and anxiety/depression.

4. Psychological status will be assessed using the Geriatric Depression Scale (short form) [36].

5. Falls risk assessment will be conducted with the Physiological Profile Assessment. This tool has been validated and includes five valid and reliable measures of physiological functioning – visual contrast sensitivity, lower limb proprioception, quadriceps strength, reaction time and postural sway. In multivariate models, weighted contributions from these five variables provide a falls risk score that can predict older people at risk of falling with 75% accuracy in both community and residential care (hostel) settings [37].

6. Additional measures of balance and mobility include the Step Test [38], co-ordinated stability test [39], Activity Measure for Post Acute Care [40] and Nottingham Extended Activities of Daily Living Index [41].

7. Participation, the individual's role in a life situation, will be measured via the Reintegration into Normal Living Index [42], Goal Attainment Scale [43], Life Space Assessment [44] and the question 'Do you get out as much as you would like?' [45].

8. Occurrence of falls will be ascertained via monthly falls calendars [46]. When a fall is recorded, the researcher will determine the time of the fall, location, mechanism and contributory factors, and whether injury has occurred, via telephone.

9. Deaths will also be recorded and verified with reference to hospital or general practitioner records.

10. Health and community service use will be recorded using monthly calendars and will be used in economic analyses.

#### Additional measures

Adherence measurements will record both the ability of health and other services to provide the recommended interventions, and the acceptance of these by the study participant.

### Treatment Allocation

#### Randomisation

Permuted block randomisation is used to achieve balanced treatment allocation [47]. A set of permuted blocks was generated for each of two strata ('frail' with 3 frailty criteria and 'very frail' with 4 or 5 frailty criteria). A random number sequence was generated for the order of treatment allocation within the blocks using the SPSS v15 RV.UNIFORM function. Varying block sizes were used. The blocks were randomly arranged within larger sized blocks. Random group allocation will be managed by project personnel not involved in measurement or intervention. The treatment allocation tables for both strata will be stored centrally off site.

#### Allocation Concealment

Enrolment of participants will be managed by the Research Consultant who will screen for inclusion criteria, seek informed consent and conduct the baseline assessment. Following completion of baseline assessment, the Research Consultant will telephone the central study office; the participant will be assigned a participant number and allocated to the intervention or control group. Staff performing the outcome assessment and data analysis will be blinded to group allocation but it is not possible to blind participants and staff administering interventions to group allocation.

### Intervention

Participants of the intervention group will receive a multidisciplinary, multifactorial intervention for one year following discharge from hospital and community rehabilitation services. The interventions will be tailored to each participant, based on their frailty characteristics assessed at baseline. Case management and weekly case conferences will facilitate coordination of the multidisciplinary delivery of the intervention.

For participants meeting the weight loss criterion, a clinical evaluation of nutritional intake at home will be made. Home delivered meals will be recommended if appropriate clinical criteria apply. In addition, if the participant's body mass index (BMI is weight in kilograms divided by height in metres squared) is < 18.5, or mid upper arm circumference is < the 10^th ^percentile (using Australian age and gender specific norms), nutritional supplementation will be offered using commercially available, high energy, high protein supplements.

If the participant reports exhaustion and the Geriatric Depression Scale score is high, consideration will be given for referral to a psychiatrist or psychologist. Where the participant is socially isolated, options will be identified to encourage greater social engagement, e.g. participation in day activity groups and telephone contact with a volunteer.

Participants classified as having grip weakness, slow four metre walk time, or low physical activity level will receive up to ten home-based physiotherapy sessions and perform a home exercise program, over the course of one year. To assist in intervention prescription, the study physiotherapist will evaluate self-efficacy using the Exercise Self-efficacy scale [48], assess stage of motivational readiness for change using the Physical Activity Stages of Change Questionnaire [49] and appraise the goal identified in the Goal Attainment Scale at baseline assessment. Findings from these assessments will be used to design a targeted, goal-focused, home-based strength, balance and endurance training regimen using the program Weight Bearing Exercise for Better Balance (WEBB) [50]. Appropriate mobility aids and other equipment will be recommended as part of this component of the intervention.

In addition, the participant's general health status will be assessed. Where appropriate, chronic disease management programs will be put in place or reinforced with the assistance of currently available health services. The principles of comprehensive geriatric assessment will be used, with careful follow-up of chronic diseases, pain and other identified conditions such as urinary incontinence. Medications will be reviewed and any apparent sub-optimal medication use will be discussed with the participant's general practitioner. Methods to encourage compliance with medications, including education about the reasons for the medication, will be provided to the participant and measures to encourage compliance with medication regimens (e.g. medication packaging in blister packs and reminder cards) will be initiated or reinforced. When the older person's family carer is noted to be experiencing significant distress due to the carer role, a supportive intervention will be provided to the carer.

Participants in the control group will receive the usual care available to older residents of Hornsby Ku-ring-gai area from their general practitioner and community services.

### Sample Size

It will be necessary to recruit approximately 230 participants, to detect a clinically and statistically significant 15% difference in lower extremity continuous summary performance score between the two groups (power = 80%, p = 0.05, dropouts = 15%, non-compliance = 15%, SD = 0.7) [26]. When 100 subjects have been recruited, means and standard deviations from baseline data will be used to check this calculation and conduct power calculations for the frailty index score.

### Statistical Analysis

The design of the study provides for three assessments with study participants – at entry, at three months, and at twelve months after the start of the intervention. Data will be coded to permit blinding to group allocation in the statistical analysis. Analysis will accord with the 'intention-to-treat' principle [51].

Prior to analysis, composite continuous scores will be created for the Fried frailty scale, using a similar methodology to the lower extremity continuous summary performance score. Between group differences in the primary outcome and secondary measures at the three month and twelve month follow-ups will be analysed using ANCOVA models where baseline values are entered as covariates. Logistic regression models will be used to compare groups on dichotomous outcomes. Non-parametric techniques will also be used where appropriate. Baseline variables will be examined, and if there are important differences between the randomisation groups these differences will be adjusted for in the models.

### Economic Analysis

Economic analysis will be carried out using similar methods to those which have been used in trials of interventions to prevent falls in older people [52-54]. The economic evaluation will take the perspective of the health and community care funder, and will include benefits measured in terms of physical functioning, hospital re-admissions, falls prevented, and utility weights derived from the EuroQOL. The total cost of the intervention and costs of health and community service utilisation and the participants' health status will be used in cost effectiveness analyses.

#### Timeframe

Recruitment commenced in January 2008. Follow-up assessment is expected to conclude in August 2011.

## Discussion

This research is an important step in the examination of an individually-targeted frailty intervention. The study's strengths are the targeting of specific domains in which the individual is frail, the multifactorial intervention which incorporates comprehensive geriatric assessment, and the robust (but pragmatic) clinical trial design.

The study methodology maximises generalisability of the results to the frail population, by facilitating recruitment and retention of frail participants, and avoiding excessive exclusion criteria. Participants are screened for frailty via a validated assessment tool, followed by the identification of the domains of frailty in which they are affected. Unlike many studies in the older population [55], most comorbidities will not cause exclusion unless they are likely to lead to less than twelve months survival. The degree of cognitive impairment deemed incompatible with involvement in this intervention was quantified as a MMSE score of 18 or less. This reflects the non inclusion of cognitive impairment in the Fried frailty criteria and the perceived difficulty of maintaining adequate adherence to the intervention if severe cognitive impairment is present.

This study is unique in that the intervention targets the domains of frailty displayed by the individual. Unlike previous randomised controlled trials, this study has the capacity to deliver a multidimensional intervention, adapted to the needs of each participant, based on comprehensive geriatric assessment. Comprehensive geriatric assessment is a multifaceted, multidisciplinary process, aiming to diagnose individuals' needs and thereby develop effective, integrated and co-ordinated management strategies [56]. Inpatient comprehensive geriatric assessment has been shown to increase the likelihood of being alive in the home at one-year follow-up [56]. This process will be facilitated by the appointment of a case manager, the availability of staff experienced in aged care, goal setting and regular multidisciplinary case conferences.

The effectiveness of exercise in the frail population is documented [22,23,57,58]. The WEBB program was chosen to deliver the exercise as it incorporates the findings of previous studies [50] as well as a recent systematic review of exercise for falls prevention (Sherrington et al., manuscript under review). Adequate intensity of exercise will be ensured by fortnightly physiotherapy home visits in the initial three months. Five visits over the subsequent nine months will enable review of the program, in light of the complex comorbidities in this population. Further, nutritional intervention in undernourished older adults has been found to increase weight and decrease mortality [59]. In frail people, nutritional supplementation has been found to increase total energy intake when used in conjunction with exercise [23].

Multiple steps have been taken to maximise adherence to the intervention. There will be a flexible time-frame for the intervention, goal setting, the involvement of family/carers, treatment in the home environment and the assessment of factors known to contribute to exercise adherence.

Applying the Fried frailty criteria in a clinical setting is not equivalent to the context in which these criteria were developed. An epidemiological study, retrospectively applying the frailty criteria to a cohort of community-dwelling adults 65 years and older, examining the prevalence of frailty and correlating frailty status with factors such as falls and hospitalisation, is clearly different to a clinical trial. It is our impression that some older people have difficulty answering the self report questions that define three of the five Fried criteria (unexplained weight loss, self-reported exhaustion, and low physical activity), and also that cognitive impairment compounds these difficulties. Other studies have since modified the original physical activity criterion [32,60] because of difficulty using the questions in the initial Fried study. The criterion used by Cesari and colleagues [32] has been adapted to fit the timeframe of this study.

There are some other potential difficulties already identified by this study in the application of the Fried criteria. The criterion of weight loss over one year does not detect significant weight loss over a longer period. With the criterion of self reported exhaustion there are some inconsistencies as some older people state that everything is an effort but they are not prepared to go on to say that they could 'not get going'. Further, some frail people with early Alzheimer's disease or other types of dementia may misinterpret the Fried criteria questions relating to self-reported exhaustion and energy expenditure. The self-report of weight loss may also be less reliable unless there is an informant present. In contrast, the Fried criteria that rely on direct functional assessment of gait speed and grip strength can be completed without difficulty.

This study uses an objective measure of frailty and therefore differs to other methods of assessing frailty that are based on clinician judgement. For example, the Canadian Study of Health and Aging Clinical Frailty Scale [1] may define older people as 'frail' somewhat differently.

If the intervention provides a clinically significant positive effect, the study could establish a new approach to the treatment of older people at risk of further functional decline and institutionalisation. In addition, an evaluation of how well the service can be provided and the participants' acceptance of the intervention will inform the extrapolation of our results to clinical practice. The intervention is readily transferable to routine clinical practice in the setting of aged care health services, and the multidisciplinary approach is relevant to multiple professional groups in aged care. If cost-effectiveness is demonstrated, the multidisciplinary intervention to decrease the rate of decline in frail people will lead to enhanced functional outcomes and more efficient utilisation of health services.

## Competing interests

The authors declare that they have no competing interests.

## Authors' contributions

This manuscript was drafted by NF and CA; PhD candidates affiliated with the Frailty Intervention Trial. KL and NM are also actively involved in the study. SK, CS, SL and IC wrote the grant application for this project that was funded by the Australian National Health and Medical Research Council in 2006. The grant application formed the basis for this manuscript, which was jointly drafted by NF and CA with all other authors contributing to its critical review and approving the final draft.

## Pre-publication history

The pre-publication history for this paper can be accessed here:


